# Cardiac Disease: Diagnosis, Treatment, and Outcomes

**DOI:** 10.3390/jpm12081212

**Published:** 2022-07-26

**Authors:** Paweł P. Rubiś

**Affiliations:** Department of Cardiac and Vascular Diseases, Jagiellonian University Medical College, John Paul II Hospital, 31-202 Krakow, Poland; pawelrub@poczta.onet.pl

Although the epidemiology—as well as the morbidity and mortality—of cardiovascular diseases (CVD) is a subject of constant change, nevertheless, CVDs are still the primary (or secondary at best after neoplasms) cause of deaths in developed countries. Moreover, it is perhaps true that the prevalence and incidence of atherosclerosis-based CVDs may be in slight decline in most developed countries, yet a far bigger wave of CVDs is looming in the quickly developing world, particularly in Asia. Thus, the main theme of this Special Issue, published recently in the *Journal of Personalized Medicine*—“Cardiac Disease: Diagnosis, Treatment, and Outcomes”—is both timely and up-to-date. This Special Issue attracted established authors from various countries/territorials and backgrounds, including Austria, Taiwan, Romania, Germany, Italy, and Poland. Altogether, there are 11 papers, including 8 original research papers, 1 systematic review, 1 state-of-the-art review, and 1 clinical case. The topics of the papers are very broad, yet all of them are within the scope of CVDs.

Without doubt, the prevention of CVDs should be the primary aim of both the medical community and the whole population, as it is a proven and the most effective population-based strategy. There are numerous ways to prevent CVDs, some of them being unique for particular countries and backgrounds. An interesting insight into morphology and function (i.e., diameters and volumetric properties) of large arteries and the heart was provided by the investigators from the KORA-MRI project, who studied 339 subjects free from CVDs with whole-body 3T-MRI scan [1]. The authors concluded that ventricular volumetric performance directly relates to vascular diameter properties [1]. Arterial hypertension, being a separate disease itself, is also one of the most important risk factors for other CVDs, such as myocardial infarction, heart failure or stroke. Despite the fact that simple lifestyle modifications and wide drug armamentarium are very effective, the number of new cases is systematically increasing and only a minority of treated patients reach the target values of blood pressure. The significance of the biological circadian rhythms of heart rate and blood pressure are being intensively investigated worldwide. It seems that too little attention is being paid towards understanding the role of biological rhythms, whereas, in fact, their preservation may be a key to success. Here, Domenico Di Raimondo and colleagues from Italy investigated four different nocturnal blood pressure profiles: dippers, mild dippers, extreme dippers and reverse dippers. We strongly encourage the reader to go through the article to find out which profile has the lowest 24 h pressure load and less cardiac remodeling [2]. Meanwhile Hsing-Yu Chen et al. from Taiwan studied median hourly ambulatory heart rate range among newly diagnosed atrial fibrillation in order to help in risk stratification. The authors’ main conclusion is that a newly-developed parameter—median hourly ambulatory heart rate range (AHRR˜24 h)—could provide an additive value to the well-known CHA_2_DS_2_-VASc score [3]. Moving towards other topic areas, there is a very interesting paper on the novel diagnostic modality of Discovery NM530c camera using 99 mTc-MIBI for the myocardial blood flow and flow reserve examination in patients with multivessel coronary artery disease. The authors analyzed the repeatability of the assessment of the whole myocardium, as well as left anterior descending, left circumflex and right coronary artery territories [4].

The last four original articles are focused on less prevalent CVDs: hypetrophic (HCM) and dilated (DCM) cardiomyopthies, myocarditis and cardiac amyloidosis. Aleksandra Karabinowska-Małocha and colleagues studied the relationships between replacement by means of late gadolinium enhancement (LGE) and interstitial (via T1-mapping) fibrosis and arrhythmic burden in HCM. The authors concluded that only the quantitative assessment of replacement fibrosis by LGE extent determines the occurrence of ventricular arrythmias in HCM, whereas the role of interstitial fibrosis in this context remains unclear [5]. Morbidity and mortality (5-year mortality of 20%) is high in DCM patients, yet there is lack of reliable tools to precisely calculate the risk. Ewa Dziewiecka et al. present the result of a longitudinal observation of a population of 735 DCM patients: 406 from the derivation cohort and 329 from the validation cohort. Each DCM patient had an individual mortality risk calculated based on the Krakow DCM Risk Score. The authors concluded that the newly-developed Krakow DCM Risk Score was found to have a good predictive accuracy and a 2-year mortality risk of >6%, discriminates well for the identification of high-risk patients and can be applied in everyday practice [6]. Despite the long history of studies on myocarditis, this issue still poses lot of questions. There are enormous variations in the management of myocarditis between centers and countries, e.g., the so-called “gold” standard—endomymocardial biopsy—is, in fact, rarely performed. Agnieszka Pawlak and co-authors have a long record of studies on myocarditis and, in the current paper, present ultrastructural changes in mitochondria in DCM patients with parvovirus B19 detected in myocardium. The authors uniquely provide the in-depth characteristics of abnormalities, detected by means of electron microscopy, in the mitochondria of DCM patients [7]. In another paper, Asan Agibetov et al. from the Medical University of Vienna explored the potential supportive role of artificial intelligence-powered algorithms applied to cardiac magnetic resonance in order to help with the diagnosis of cardiac amyloidosis [8].

There are also two very interesting review papers in the Special Issue. Infective endocarditis (IE) and, especially, one of its subtypes—cardiac device-related infective endocarditis—is a very serious disease with high morbidity and mortality. The accurate diagnosis of IE is of paramount importance as it determines further management, including surgery, yet it is a difficult and debatable subject. Katarzyna Holcman and co-authors present a high-quality systematic review on the diagnostic value of 99 mTc-HMPAO-labelled white blood cell scintigraphy and 18F-FDG PET/CT in cardiac device-related infective endocarditis. This paper analyses all published data on scintigraphy and PET in the setting of cardiac device-related infective endocarditis [9]. Another review comes from an established group with a long record of studies in the field of aortic stenosis; in this paper, Joanna Natorska et al. comment on current randomized trials investigating the medical treatment of aortic stenosis, including strategies based on lipid-lowering and antihypertensive therapies, phosphate and calcium metabolism, and novel therapeutic targets such as valvular oxidative stress, coagulation proteins, matrix metalloproteinases and the accumulation of advanced glycation end products [10]. The final paper of this Special Issue is a case report on a rare coexistence of endocarditis in Behçet’s disease. The authors provide a thorough chronological description of events and a detailed characterization of Behçet’s disease [11].

All the articles were subjected to detailed peer review and represent high quality papers, published in the *Journal of Personalized Medicine*. We strongly encourage all potential readers to become familiar with this Special Issue.
